# Cloning, expression and purification of a polytopic antigen comprising of surface antigens of *Toxoplasma gondii*

**Published:** 2017-08

**Authors:** Abbas Alibakhshi, Mojgan Bandehpour, Bahram Kazemi

**Affiliations:** 1 Department of Biotechnology, School of Advanced Technologies in Medicine, Shahid Beheshti University of Medical Sciences, Tehran, Iran; 2 Cellular and Molecular Biology Research Center, Shahid Beheshti University of Medical Sciences, Tehran, Iran; 3 Department of Biotechnology, School of Medicine, Shahid Beheshti University of Medical Sciences, Tehran, Iran

**Keywords:** *Toxoplasma gondii*, Polytopic antigen, SAG, Epitope mapping

## Abstract

**Background and Objectives:**

Polytopic antigens are recently applied for replacing crude antigens, for control of infectious agents. The surface of the Toxoplasma is covered with immunogenic antigens namely surface antigens (SAGs). These antigens possess several immunogenic epitopes, inducing immune responses.

**Materials and Methods:**

In this study, a DNA construct comprising of sequences encoding epitopes from SAG1, 2 and 3 was designed and cloned into pET28a expression vector and subsequently expressed and purified, using Ni-NTA column.

**Results:**

The SDS-PAGE and Western blotting analysis showed that polytopic genes were successfully expressed and purified.

**Conclusion:**

The surface antigenic protein of *T. gondii* can be applied in the future epitope-based applications.

## INTRODUCTION

*T. gondii* is a ubiquitous intracellular protozoan parasite that infects a wide range of warm-blood animals, including humans. Its complex life cycle involves definitive and intermediate hosts. It contains asexual phase in all hosts, including a sexual phase in the gut of the cat (1). Cats are the only definitive host. This obligate parasite causes death in immunocompromised and immusuppressed individuals, as well as in utero. This infection is costly to take care of and causing distress and significant problems for livestock husbandry. Therefore, controlling approaches can minimize the parasite’s harm to people. *T. gondii* possesses several antigens, which have been much used for designing a vaccine and prompt diagnosis (1, 2).

The surface of the parasite is covered with a type of glycosylphosphatidyl inositol (GPI)-anchored antigens, belonging to the surface antigens (SAGs) family (3). SAG1 is one of the crucial immunodominant ligands in the invasion of the parasite into host cells. SAG1 has been demonstrated to be an important protein, in developing subunit vaccines and rapid diagnostic tests (4). SAG2 is another attachment factor on the surface of the parasite, which is a serologically immunodominant protein. It has been shown that it is relevant in the host-parasite interface and interacts with both distinct innate and adaptive immune mechanisms (5). SAG3, a surface protein similar to SAG1, in structure and associated with binding to the host cells. Heparin sulfate proteoglycan mediate attachment of the parasite to the host cell surface (6).

In the course of the acute disease, some of the SAG family members are clearly immunodominant and induce a strong immune response against the parasite, during early stages of infection. The surface antigens are anchored to the plasma membrane and it appears that have significant exposed epitopes for immune systems, playing an important role in stimulation of host immune response and thereby can be used for applications of diagnosis and vaccine development (1, 7). Herein, we selected the strongest epitopes of three surface antigens of *T. gondii*, in view of better stimulation of human immune system by *in silico* methods and constructed a DNA sequence, based on the indicated epitopes for the production of a poly-epitope protein. This protein was then expressed and purified, in order to be used for different medical applications.

## MATERIALS AND METHODS

### Polytopic construct and plasmid.

The B-cell epitopes of the three proteins were selected based on Emini surface accessibility, Kolaskar and Tongaonkar antigenicity, Parker hydrophilicity, using the IEDB (Immuno Epitope Database) server website (8). The T-cell agretope was also selected, using the IEDB server website. The amino acid sequence was selected, so that both B-cell epitopes and T-cell agretopes are included. Three sections from three proteins were joined in developing a 135 amino acid sequence and then was reverse translated into a DNA sequence based on human codon usage. pQE30 vector was synthesized by Generay Biotech Co., and the polytopic constructs was inserted between *SacI* and *HindIII* site of pQE30 vector.

### Cloning of polytopic construct into pET28a vector.

Multiple cloning site was amplified from pQE30/polytopic genes, using PCR with universal primers (Forward: 5′-AAATGGGCGGTAGGCGTG-3′ and Reverse: 5′-GCACCACCCCGGTGAACAG-3′). Both the resulting product and pQE30/polytopic genes were double digested with *SacI* and *HindIII* to release polytopic sequence and pET28a vector was also treated with *SacI* and *HindIII* to linearize the supercoiled plasmid. The product PCR and linear peET28a vector were then treated with T4 ligase for ligation of polytopic construct into bacterial expression pET28a vector. The vector was finally transformed into TOP10 *E. coli* strain. The colony-PCR was performed for selection of ligated vectors. PCR of the multiple cloning site of pET28a, using universal primers (Forward: 5′-CGAGCCCGATCTTCCCCATC-3′ and Reverse: 5′- GCTAGTTATTGCTCAGCGG-3′) and sequencing analysis was used for confirmation of ligation.

### Expression of recombinant polytopic His6.

The BL21 (DE3) *E. coli* were cultured in Luria-Bertani (LB) broth medium, supplemented with kanamycin, at 37°C with a shaking speed of 200 rpm. The cultured cells were transformed with ligated pET28a vector. The transformed cells were induced when OD600 of growing cells reached 0.6–0.8, by the addition of isopropyl-β-D-1-thiogalactopyranoside (IPTG) in a final concentration of 1 mM for 2–5 hours. The cells were harvested by centrifugation at 8000rpm for 5 min. The induced cells were lysed in lysis buffer using sonication. SDS-PAGE and Western blotting were used to detect the induced polytopic-His6 protein. SDS-PAGE was performed using a 12% polyacrylamide gel. The proteins were mixed with 2X SDS-loading buffer (100 mMTris-HCl pH6.8, 20% glycerol, 4% SDS, 0.005% bromophenol blue and 200mM DTT) and heated at 85°C for 5 min and applied for gel electrophoresis (9). After this, the gel was stained with coomassie blue R-250 and then destained. For Western blotting, after gel electrophoresis and after separation of proteins by SDS-PAGE, the proteins were transferred to a nitrocellulose membrane. The membrane was then blocked with 3% skimmed milk powder in phosphate buffer saline (PBS) for one hour. After washing the membrane three times using PBS with 0.1% Tween 20 (PBST), membrane was incubated with anti-polyHistidine–Alkaline Phosphatase (ALP) antibody (1:10000 in PBS), at a shaking speed of 40 rpm for 2 hours. Subsequently, the membrane was washed twice in PBS and once in PBST. The target protein was detected by adding alkaline phosphatase reaction substrates: bromochloroindolyl phosphate (BCIP) and nitrobluetetrazolium (NBT).

### Purification polytopic His6 protein.

After induction of more cells with IPTG for 3 hours, the cells were lysed in equilibrium buffer (2M urea, 20 mMTris-HCl, 500mM NaCl, 50mM Imidazole, 0.5% triton X-100 and PMSF, pH 8), pelleted and homogenized by sonication. The lysate was then centrifuged at 5000 rpm for 20 min and loaded on a Ni(II)-immobilized metal-affinity chromatography (10). The proteins can be purified due to high affinity histidine residues to Ni(II) ions. After shaking the nitrilotriacetic acid (Ni-NTA) column, the column was washed for three times with washing buffer (0.4 M urea, 20 mMTris-HCl, 500mM NaCl, 50 mM Imidazole, 0.5% triton X-100 and PMSF, pH 8). Finally, the proteins were eluted with elution buffer (0.4 M urea, 20 mMTris-HCl, 500mM NaCl, 500mM Imidazole, pH 8). Western blotting was also used to detect the purified polytopic-His6 protein, as described above.

### Analysis of purified polytopic protein.

The sequence of polytopic protein was also confirmed, by comparing against Basic Local Alignment Search Tool (Blast) software. The sequence was also analyzed, in regards to physicochemical properties, such as polarity of amino acids.

## RESULTS

### Preparation and cloning of polytopic genes.

**Fig. 1.**
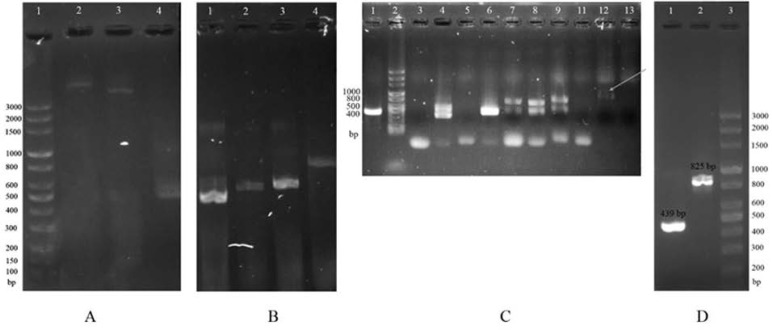
Preparation and cloning of polytopic genes. (A) Preparation of polytopic genes. Lane 1: 3kb DNA ladder; Lane 2: pET28a vector; Lane 2: pQE30 vector; lane 4: PCR product of the multiple cloning site of pQE30/polytopic genes, using universal primers of pQE30. (B) Digestion of vectors with *HindIII* and *SacI*. Lane 1: Double digestion of pQE30/polytopic gene; Lane 2: Intact pQE30/polytopic genes; Lane 3: Double digestion of pET28a; Lane 4: Intact pET28a. (C) Colony-PCR. Lane 1: PCR product of pET28a using universal primers of pET28a; Lane 2: 3kb DNA ladder; Lanes 3–13: PCR product of pET28a/polytopic genes, using universal primers of pET28a. (D) Confirmation of gene cloning, using amplification of the gene by PCR on the vector. Lane 1: PCR product of pET28a using universal primers; Lane 2: PCR product of pET28a/polytopic genes vector, using universal primers; Lane 3: 3kb DNA ladder.

**Fig. 2.**
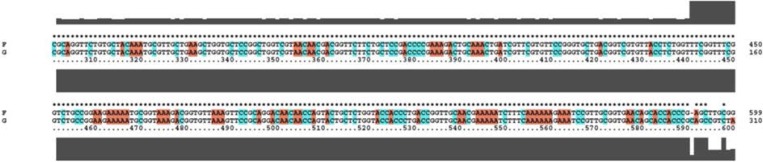
Sequencing analysis of ligation of polytopic genes into *SacI* and *HindIII* sites of pET28a vector.

The resulting sequence had a length of 405 base pairs (bp) (Fig. 1A). The pET28a vector was successfully prepared for insertion of the polytopic genes (Fig. 1B) and after colony-PCR from 11 colonies, the PCR product of colony No. 9 (lane 12, Fig. 1C) was selected for restriction enzyme reaction and subsequent ligation into linear pET28a. The ligation was successfully performed, as is shown in Fig. 1D, as a PCR product of approximately 825 bp (using the universal primers for pET28a/polytopic genes should produce a product of 825 bp) (Fig. 1D). Moreover, the sequencing analysis confirmed the success ligation of polytopic genes into *SacI* and *HindIII* sites of pET28a vector (Fig. 2).

### Expression of polytopic genes.

**Fig. 3.**
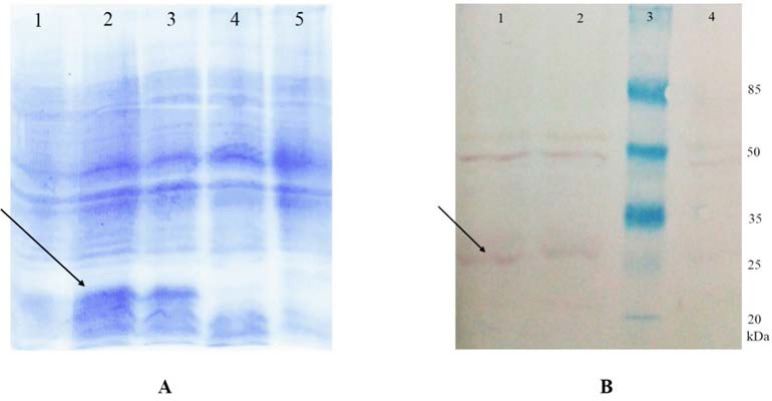
Expression of polytopic genes from *E. coli.* (A) SDS-PAGE analysis of protein extracts. Lanes 1, 2 and 3: Protein extracts of *E. coli* cells transformed with pET28a/polytopic genes, 5, 3 and 2 hours after induction, respectively; Lane 4: Protein extracts of *E. coli* cells transformed with pET28a/polytopic genes before induction; Lane 5: Protein extracts of *E. coli* cells. (B) Western blotting analysis of protein extracts. Lanes 1 and 2: Protein extracts of *E. coli* cells transformed with pET28a/polytopic-His6, 3 and 2 hours after induction, respectively; Lane 3: Protein molecular size marker; Lane 4: Protein extracts of control *E. coli* cells.

The SDS-PAGE analysis showed that gene expression is induced for all three periods of 2, 3 and 5 hours after the induction (Fig. 3A). The results showed multimer forms of polypeptide (14kDa) on the SDS-PAGE and Western blot membrane that it is related to the polypeptide structure (Fig. 3B).

### Purification of polytopic protein-His6.

**Fig. 4.**
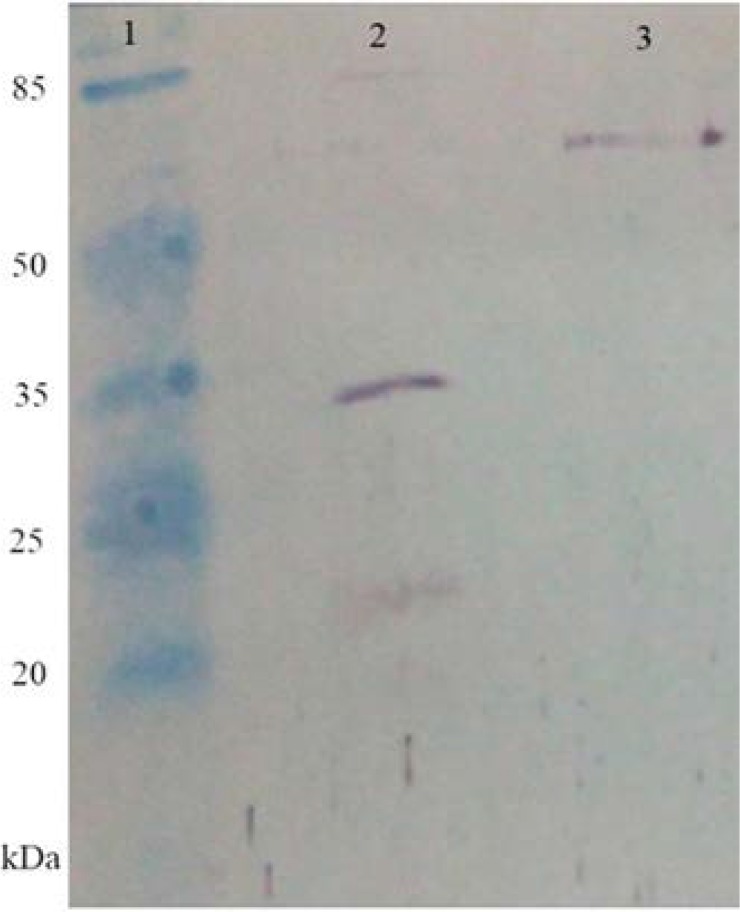
Purification of polytopic protein-His6. Western blotting analysis of purified protein. Lane 1: Protein molecular size marker; Lane 2: Protein extracts of *E. coli* cells transformed with pET28a/polytopic genes, 3 hours after induction; Lane 3: Purified protein.

The protein seems that is expressed as multimer and so in the purification processby Ni-NTA affinity column chromatography, it was in the eluted fraction, as multimer proteins (Fig. 4).

## DISCUSSION

In the current study, a polytopic protein was designed and produced for controlling strategies, such as vaccine design and diagnostic methods against toxoplasmosis. The surface antigens (SAGs) of *T. gondii* were selected for designing the polytopic protein. The studies show that these antigens have good potential for stimulating the immune system against the parasite. The SAGs possess many immunogenic regions and in this study, an antigenic protein containing three regions with high immunogenic potential from three SAGs (SAG1, 2 and 3) was designed and successfully purified, using affinity chromatography.

The designing strategies for immunostimulatory polytopic proteins has been shown that can be effective for inducing an immune response against the original proteins, derived from infectious agents, protecting the vaccinated host against the infection. A large number of *T. gondii* vaccine design strategies have been made in the past years to control infection and the vaccine development is ongoing (11). The micro-organisms such as *T. gondii* has a complex structure, representing in numerous antigens and thus different antigens may cause infection in individuals (12). In addition, the complete protein antigens do not show immunogenic functions, whereas specific regions of proteins such as epitopes are responsible for inducing immune response (13). Therefore, immunization with a vaccine comprising of T-cell agretopes and B-cell epitopes of different antigens, presumed to be more effective in stimulating an immune response. The various advantages of these approaches leads to the application of polytopic vaccines, as an attractive and promising tool for enhancing protective immunity against different disorders. Multi-epitopic proteins have also been employed in the development of new diagnostic methods in the recent years. Although serological tools are the primary assays for diagnosis of *T. gondii*, but the crude native antigens used in the current diagnostic methods do not exhibit proper sensitivity and specificity. Multi-epitopic protein based identification approach, using bioinformatics tools expected to be easier to use for standardization and diagnosis of the infection with more sensitivity and accuracy. Hajissa et al. designed a single gene, encoding the potential epitopes of three antigens of *T. gondii* (SAG1, GRA2 and GRA7) and after cloning into an expression vector, the expressed and purified protein was used for evaluation of immunoreactivity of the synthetic protein, using human sera. The ELISA analysis showed high immunoreactivity of multiepitopes in serodiagnosis of anti-toxoplasma antibodies with 100% sensitivity and specificity (14).

The success of this strategy is based on rational design of polytopic structure and sequence, protein expression and purification, using ideal and appropriate techniques. Here, we designed a polytopic genes encoding immunogenic regions of SAG1, 2 and 3, using *in silico* tools. The polytopic genes sequence was cloned into an expression vector and successfully expressed and purified. This antigenic protein can be used in the epitope-based applications such as vaccine development and new diagnostic kits for toxoplasmosis.
